# An audit to assess physical health monitoring of patients following their admission to the general adult psychiatric inpatient wards in Mersey Care NHS Foundation Trust

**DOI:** 10.1192/bjo.2021.260

**Published:** 2021-06-18

**Authors:** Declan Hyland, Agatha Milner, Ellen Carter

**Affiliations:** 1 Consultant Psychiatrist, Clock View Hospital, Liverpool, Mersey Care NHS Foundation Trust; 2 5th year medical undergraduate, University of Liverpool

## Abstract

**Aims:**

This audit aimed to establish whether patients undergo physical health monitoring within 24 hours of admission to one of the general adult inpatient wards in Mersey Care NHS Foundation Trust, as per Trust policy.

**Background:**

Mean life expectancy in individuals with severe and enduring mental illness (SMI) is 15-20 years shorter than that of the general population. A significant proportion of excess mortality in patients with SMI is due to natural causes, e.g. cardiovascular disease and type II diabetes mellitus. Although SMI patients are at greater risk of developing chronic physical health problems, they often receive worse health care than the general population. Shared care of SMI patients between primary and secondary healthcare professionals causes uncertainty over who is responsible for monitoring the physical health of these patients.

**Method:**

A list of all inpatients on the eight general adult wards in the Trust was obtained in September 2020, producing a sample of 135 inpatients.

An audit tool was designed, capturing demographic data – gender, age, ethnicity. The patient's psychiatric diagnosis was recorded. The tool captured whether each of the following were measured following admission – body mass index (BMI), blood pressure (B.P), serum cholesterol level, QRISK score and HbA1c level, and, if so, whether this was done within 24 hours of admission. For those patients who were smokers, being offered nicotine replacement therapy was documented.

**Result:**

Of the 135 inpatients, 10 didn't have any physical health monitoring completed and were excluded from the sample, making the final sample 125 inpatients. 68 of the inpatients were male, 57 were female. 98 had a diagnosis of an SMI, 27 did not. Most inpatients were of “white British” ethnicity. 91% of the sample had a BMI measured within 24 hours of admission, but only 62% had a B.P done, 59% had a serum cholesterol level done and 58% had an HbA1c level done within 24 hours of admission. 78% of eligible patients had a QRISK score calculated. 97% of inpatients who were smokers were offered nicotine replacement therapy, but only 13% accepted it.

**Conclusion:**

The majority of patients admitted to the general adult inpatient wards have an SMI. The audit findings show need for improvement in physical health monitoring following admission. Creation and implementation of a checklist of physical health parameters to be measured within 24 hours of admission could help improve performance. Use of motivational interviewing may help increase uptake of nicotine replacement therapy in smokers.

